# Efficacy of WHO recommendation for continued breastfeeding and maternal cART for prevention of perinatal and postnatal HIV transmission in Zambia

**DOI:** 10.7448/IAS.18.1.19352

**Published:** 2015-07-01

**Authors:** Mary S Ngoma, Amita Misir, Wilbroad Mutale, Emmanuoil Rampakakis, John S Sampalis, Angela Elong, Sam Chisele, Abel Mwale, Jonathan K Mwansa, Scholastica Mumba, Mula Chandwe, Richard Pilon, Paul Sandstrom, Samantha Wu, Kristen Yee, Michael S Silverman

**Affiliations:** 1 Department of Paediatrics and Child Health, University of Zambia, Lusaka,, Zambia; 2 Department of Pediatrics, Western University, London, Canada; 3 Department of Public Health, University of Zambia, Lusaka, Zambia; 4 Department of Epidemiology and Biostatistics, McGill University, Montreal, Canada; 5 JSS Medical Research, Montreal, Canada; 6 Department of Medicine, University of Toronto, Toronto, Canada; 7 Department of Obstetrics and Gynecology, University of Zambia, Lusaka, Zambia; 8 Department of Anesthesiology, University of Zambia, Lusaka, Zambia; 9 Department of Paediatrics, Arthur Davison Children's Hospital, Ndola, Zambia; 10 Department of Nursing, Chelstone Clinic, Lusaka, Zambia; 11 Public Health Agency of Canada, Ottawa, Canada; 12 Harvard Medical School, Cambridge, Mass; 13 Grand Challenges, Ottawa, Canada; 14 Division of Infectious Diseases, Western University, London, Canada

**Keywords:** breastfeeding transmission of HIV, vertical transmission, option B+, infant survival, efficacy of WHO guidelines, perinatal transmission of HIV

## Abstract

**Introduction:**

To prevent mother-to-child transmission (MTCT) of HIV in developing countries, new World Health Organization (WHO) guidelines recommend maternal combination antiretroviral therapy (cART) during pregnancy, throughout breastfeeding for 1 year and then cessation of breastfeeding (COB). The efficacy of this approach during the first six months of exclusive breastfeeding has been demonstrated, but the efficacy of this approach beyond six months is not well documented.

**Methods:**

A prospective observational cohort study of 279 HIV-positive mothers was started on zidovudine/3TC and lopinavir/ritonavir tablets between 14 and 30 weeks gestation and continued indefinitely thereafter. Women were encouraged to exclusively breastfeed for six months, complementary feed for the next six months and then cease breastfeeding between 12 and 13 months. Infants were followed for transmission to 18 months and for survival to 24 months. Text message reminders and stipends for food and transport were utilized to encourage adherence and follow-up.

**Results:**

Total MTCT was 9 of 219 live born infants (4.1%; confidence interval (CI) 2.2–7.6%). All breastfeeding transmissions that could be timed (5/5) occurred after six months of age. All mothers who transmitted after six months had a six-month plasma viral load >1,000 copies/ml (p<0.001). Poor adherence to cART as noted by missed dispensary visits was associated with transmission (p=0.04). Infant mortality was lower after six months of age than during the first six months of life (p=0.02). The cumulative rate of infant HIV infection or death at 18 months was 29/226 (12.8% 95 CI: 7.5–20.8%).

**Conclusions:**

Maternal cART may limit MTCT of HIV to the UNAIDS target of <5% for eradication of paediatric HIV within the context of a clinical study, but poor adherence to cART and follow-up can limit the benefit. Continued breastfeeding can prevent the rise in infant mortality after six months seen in previous studies, which encouraged early COB.

## Introduction

The Joint United Nations Programme on HIV/AIDS (UNAIDS) has set a global plan to reduce HIV infections among HIV-exposed children to below 5% by 2015 and to keep their HIV-positive mothers alive through the provision of antiretroviral medication [1]. Despite the risk of mother-to-child transmission (MTCT) of HIV, in many developing countries, no acceptable, feasible, affordable, sustainable or safe alternative to breastfeeding exists [2–4]. Reaching the 5% target will require prevention of antepartum, intrapartum and postpartum transmission.

In the absence of antiretroviral prophylaxis, MTCT is lower during the first six months of life in exclusively breastfed (EBF) infants compared to those who mixed feed [5, 6]. Multiple studies have shown that maternal combination antiretroviral therapy (cART) can reduce postpartum MTCT for up to six months of EBF [7–9].

In HIV-exposed children, infant mortality and morbidity (e.g. failure to thrive (FTT), diarrhoea, pneumonia, malaria and tuberculosis) are increased after early cessation of breastfeeding (COB) (at 4–6 months) [9–13]. Therefore, in 2010 the World Health Organization (WHO) issued new recommendations which include EBF for 6 months followed by complementary feeding (CF) (defined as continued breastfeeding with the addition of solid or semi-solid foods) to 12 months of age and followed by gradual COB [2, 14]. Maternal cART is recommended during pregnancy and postpartum breastfeeding to prevent MTCT (PMTCT). For women whose CD4 cells are <500 cells/µl, on-going maternal cART after COB is recommended to protect the mothers health [15]. For women whose CD4 >500 cells/µl, the WHO recommends either option B where maternal cART is discontinued after COB or Option B+ where maternal cART is continued indefinitely [15]. Unfortunately, there are only limited data on the impact of maternal cART on infant adverse events and PMTCT while breastfeeding beyond six months of age. Therefore, the quality of the supporting evidence for the new WHO guidelines is still noted as low to moderate [2, 14, 15]. We, therefore, carried out a study to assess the efficacy and safety of continued breastfeeding while the mother receives cART.

## Methods

This was a prospective observational cohort study. Recruitment took place from December 2008 to December 2009 at the Chelstone Public Clinic in Lusaka, Zambia.

Inclusion criteria were: age ≥15 years, HIV seropositivity, confirmed pregnancy with the ability to initiate cART at 14–30 weeks gestation, intention to EBF for six months, ability to give informed consent and to attend follow-up visits. Exclusion criteria were previous cART (other than single-dose nevirapine or ≤1 month of zidovudine for PMTCT prophylaxis, which were not contraindications), known major illness likely to influence pregnancy outcome or place the participant at increased risk for adverse events from cART, including diabetes, severe renal, liver or heart disease, active tuberculosis, severe anaemia (Hgb <8 g/dl) or continuing therapy with medications, which are contraindicated for co-administration with ritonavir/lopinavir.

All mothers received zidovudine (ZDV) 300 mg/lamivudine (3TC) 150 mg (Combivir^®^, ViiV healthcare, Middlesex, UK) bid and lopinavir 200 mg/ritonavir 50 mg (trade name Aluvia^®^ in Zambia, Abbvie, Chicago, IL) two tablets bid initiated between gestational age (GA) 14 and 30 weeks, and continued during labour, breastfeeding and COB. Women were enrolled in the national cART programme upon COB and were continued indefinitely on cART regardless of CD4 count (as per WHO option B+). Lopinavir/ritonavir was used, as efavirenz was not recommended during pregnancy at the time of this study [16], and nevirapine was contraindicated for the majority of women due to high baseline CD4 [17]. This approach allowed rapid initiation of therapy prior to CD4 results. Infants received oral ZDV for five days postpartum. EBF was encouraged to 6 months, with CF starting at 6 months and continued to 12 months, followed by the recommendation to cease breastfeeding between 12 and 13 months. Women received on-going counselling regarding medication adherence, text message reminders for clinic visits and stipends for food and transport (30,000 kwacha, equivalent to ~$6US/visit). Mothers were screened antepartum for syphilis using a non-treponemal test and were given multivitamins. Sexually transmitted diseases were diagnosed and treated syndromically as per national guidelines. Mothers received counselling on infant nutrition. GA dating was based on recall of last menstrual period. Follow-up visits took place antepartum at 4 weeks after enrolment, 36 weeks gestation; at birth; at postpartum infant ages 2, 6 weeks and 3, 6, 9, 12, 15 and 18 months. Infant dried blood spots for PCR were obtained at all postpartum visits. Infant survival data were also obtained at 24 months. Maternal plasma viral loads and drug resistance genotyping were assayed on all transmitting mother-infant pairs using standard techniques [18, 19] (see Supplementary files). Maternal plasma viral load was also assayed at birth and six months on a convenience sample of 96 non-transmitting mothers.

Standard breastfeeding definitions were used for the study and were obtained from UNICEF [20]:

*Exclusive breastfeeding (EBF):* Only breast milk+medicines/vitamins.

*Mixed feeding:* Breast milk and non-human milk (including formula) and/or semi-solid/solid foods before six months.

*Complementary feeding (CF):* Continued breastfeeding with the addition of solid or semi-solid foods beyond six months.

*Replacement feeding:* No breast milk at all, fed only non-human milk or semi-solids/solids.

Ethical approval for the study was obtained from the research ethics boards of the University of Zambia in Lusaka and also Lakeridge Health Corporation in Canada. All mothers provided written informed consent. The study funders had no role in the design of the protocol, conduct of the study, the analysis of data or the writing of the manuscript.

### Statistical methods

The primary outcome of interest was the rate of HIV-free survival after an extended period (i.e. 12 months) of breastfeeding followed by gradual COB. Secondary outcomes of interest included HIV transmission rates and mortality particularly as they corresponded to the different time periods of exclusive breastfeeding (0–6 months), CF (6–12 months) and COB (12–18 months). The incidence of HIV transmission and death was described with the corresponding incidence rates up to 18 months post-partum for the above time periods. Denominators for these incidence rates include only those infants who remained at risk for transmission (i.e. were known to be HIV-uninfected and surviving) at the beginning of each relevant time period.

For all analyses, patients who were lost to follow-up were not included in the analysis for the periods when they were not available. This was decided in order to prevent these patients from being inadvertently considered as non-transmitting or surviving and thus artificially inflating efficacy.

Categorical variables were summarized using frequency distributions and percentages, whereas continuous variables were described with the mean and standard deviation. We used independent samples t-test to assess statistical differences in (i) maternal age, maternal CD4 at birth and infant GA at birth between women lost to follow-up postpartum and those completing 24 months of follow-up and (ii) mean viral load between transmitting and non-transmitting mothers. We used Pearson's chi-square test to assess statistical differences in (i) the viral load categories at six months between transmitting and non-transmitting mothers; (ii) infant mortality based on presence or absence of low birthweight and (iii) HIV transmission between women missing vs. not missing a dispensary visit. Changes in viral load and viral load detection over six months of treatment were assessed with the paired *t*-test and the McNemar test, respectively. The incidence of HIV transmission and death was described with the incidence rate and the incidence rate ratio and the corresponding 95% confidence intervals (95% CI). A comparison of different time intervals with respect to these outcomes was performed using the person-time chi-square (χ^2^_P-T_) test [21, 22].

Predictors of adverse event count by time interval were investigated using a saturated Poisson regression model where time interval and individual person follow-up time were incorporated as adjusting covariates. Given that this was a saturated model, goodness of fit was not assessed. Over dispersion was examined by dividing the Pearson statistic by its degrees of freedom and was corrected, as required, by inflating accordingly the covariance matrix.

All analyses were performed using SAS 9.2 (SAS Institute, Cary, NC), and all analyses were performed using a 0.05 significance level.

## Results

### Study population

Clinical baseline characteristics of the cohort are presented in Table 1.

**Table 1:** Maternal baseline characteristics (live born infants only)

Maternal baseline characteristics	Available *n*	(*N*=231)
Maternal age, years, mean (SD)	226	28.1 (5.5)
Gestational age (GA) at birth, weeks, mean (SD)	229	38 (3.5)
Sexually transmitted infections (STIs), n (%)	225	41 (18%)
Multiple birth, n (%)	225	7 (3%)
CD4 <350 at study entry, n (%)	209	125 (60%)
CD4 <200 at study entry, n (%)	209	45 (22%)
CD4 count, mean (SD)	209	353.8 (207.1)
Anaemia (haemoglobin <10 g/dl), n (%)	225	55 (24%)

*Note*: Detailed socio-demographic data are available in Supplementary Table 1.

### Transmission

**Figure 1:**
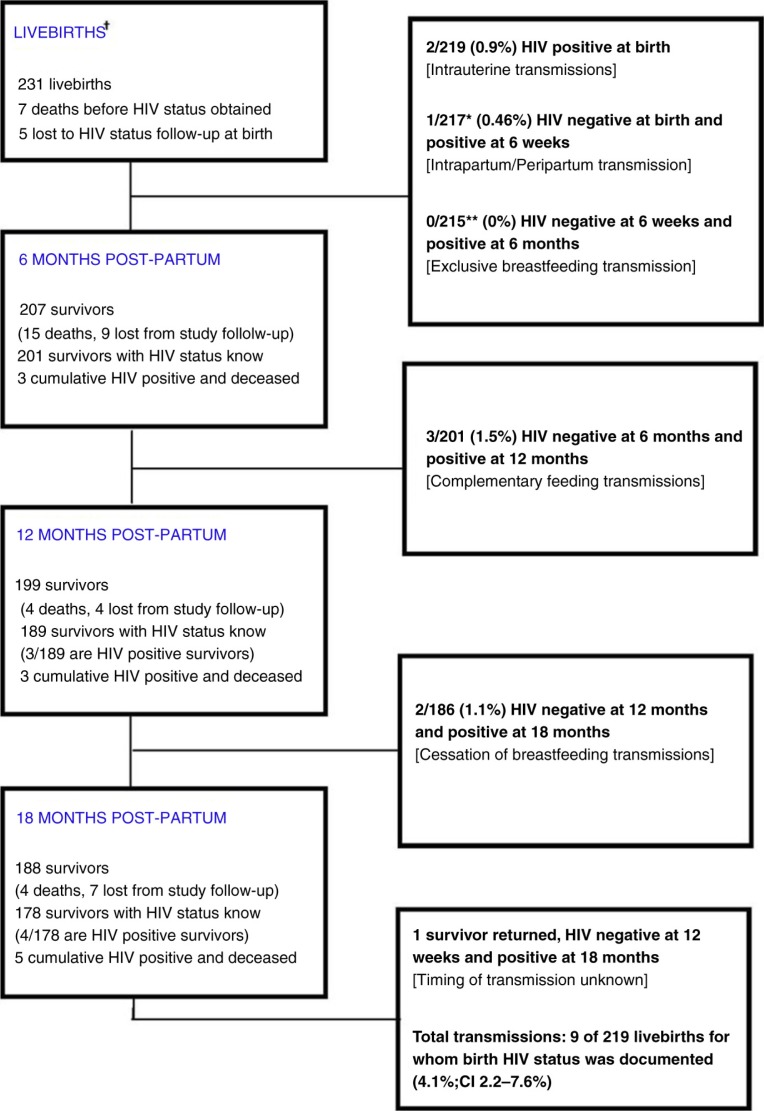
Transmission status follow-up on livebirths and survivors. ^†^Three hundred and eighty-four pregnant women were approached, of which 80 were not eligible (due to presentation outside of 14–30 weeks gestation) and 25 declined enrolment, leaving 279 women who were recruited. Of the 279 enrolled women, 45 of 279 (15%) defaulted before birth, leaving 234 women. Of these 234 women, 8 had stillbirths and 226 had livebirths. These 226 mothers delivered 231 liveborn infants (five pairs of twins were included). *Two infants HIV positive at birth so no longer at-risk for transmission at start of time interval, therefore removed from denominator. **Infant became HIV positive+1 infant died between birth and 6 weeks, so no longer at-risk for transmission at start of time interval, therefore removed from denominator.

Transmission status at follow-up is presented in Figure 1. Overall, when considering all 231 live births, HIV status was known at 18 months or at the last scheduled test interval before their death (whichever came first) for 201/231 (87.0%). There was no difference between the women who were lost to follow-up postpartum and those who completed 24 months of follow-up when compared by maternal age (p=0.55), maternal CD4 at birth (p=0.89) or infant GA at birth (p=0.946). A total of 9/219 (4.1%; 95% CI: 2.2–7.6%) infants acquired HIV during the study period. There was a trend towards a higher transmission rate/100 person-weeks during 6–18 months (0.0529/100 person-weeks) when compared to six weeks to six months (0/100 person-weeks; p=0.167).

Details of the individual transmissions are found in Supplementary Table 2. Baseline maternal CD4 count (<200 vs. >200; odds ratio, OR <350 vs. >350, OR <500 vs. >500) was not correlated with infant mortality or postpartum transmission (data not shown).

### Treatment failure and late HIV transmission

Five of five women who transmitted after six months had a plasma viral load (VL) >1,000 at six months (range 3,252–459,866; mean 4.75 log_10_, SD 0.82). In comparison, mean viral loads at six months in the 96 non-transmitting mothers tested were lower (mean 0.61 log_10_ SD 1.4; p<0.0001). Twelve of 96 (12.5%) non-transmitting mothers had a VL >1,000 (p<0.001) and 16/96 (16.7%) had a VL >50 at six months (p<0.001). Including all transmitting and non-transmitting mothers, more mothers had a detectable VL (>50) at birth (40 of 99 (40.4%)) than at six months (p=0.002), and mean Log_10_ viral load at six months (1.21, SD 1.94) was not higher than at birth (1.25, SD1.73; p=0.86). Data available on seven of nine transmissions showed one transmission (antepartum) to be associated with resistance to the cART regimen (K65R in mother and infant; Supplementary Table 3).

### Mortality

Four of 279 (1.4%) mothers died including one maternal death with accompanying miscarriage/foetal death. Details of individual infant and maternal deaths are given in Supplementary Tables 4 and 5.

Two hundred and eleven of 231 (91.3%) live born infants had 24-month survival data, the remainder being lost to follow-up. Cumulative postnatal mortality was 25/231 live births (10.8%; 95% CI: 6.8–14.8%). Data on cumulative rate of HIV infection and/or death were available for 226 infants with a cumulative rate of 29/226 (12.8%; 95% CI: 7.5–20.8%) (includes 20 deaths of HIV-uninfected infants, 5 deaths in HIV-infected infants and 4 transmissions in surviving infants).

HIV-uninfected infant death rate was highest in the first six months of life, falling during the second six months with no evidence of any rise after COB at 1 year (Table 2).

**Table 2:** Death incidence rate ratio by time to event for infants who were HIV uninfected

Time interval (month)^a^	Number of deaths	Incidence rate (per 100 person-week)	Incidence rate ratio	95% CI of IRR	*p*
(0–6)	12	0.2346	1.0000	NA	NA
(6–12)	4	0.0838	0.3570	(0.1148, 1.1102)	0.03952
(12–18)	2	0.0433	0.1847	(0.0412, 0.8284)	0.00160
(18–24)	2	0.0457	0.1947	(0.0434, 0.8735)	0.00228

a *Note*: Patients contributed the respective person-time corresponding to each time period provided they were known to be remaining at risk of death during that time period. Therefore, those who were lost to follow-up at any point did not contribute person-time for subsequent time periods. Mortality at each time interval is compared with that at the first time interval (0–6 months). Confidence intervals were determined considering a Poisson distribution for the number of deaths. NA, not applicable.

Using the same intervals as in the recent BAN study, which showed an increase in HIV-uninfected infant mortality with COB at 28 weeks [9], HIV-uninfected infant mortality in our cohort fell from 0.2183/100 person-weeks (95% CI: 0.1162–0.626) during the first 28 weeks of life to 0.0755/100 person-weeks (95% CI: 0.0156–0.2016) during weeks 29–48 (incidence rate ratio =0.346 (95% CI: 0.0983–1.22); p=0.04).

Total mortality (HIV-infected and -uninfected infants) was also higher in the first six months of life 0.2839/100 person-weeks (95% CI: 0.1589–0.4564) than between 6 and 12 months 0.0813/100 person-weeks (95% CI: 0.022–0.1933; incidence rate ratio=0.286 (95% CI: 0.069, 0.8989) (p=0.02)).

### Maternal adherence

**Figure 2:**
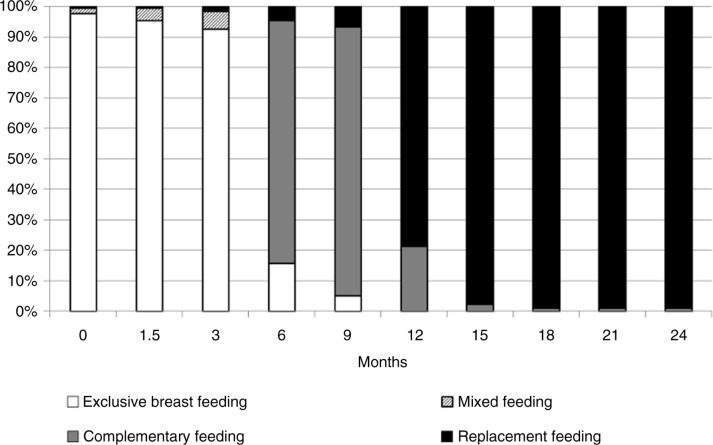
Type of feeding over time. Note: definitions for feeding types [24].

Self-reported adherence to breastfeeding recommendations was requested at each visit with full data to COB or death being available on 205/231 infants (88.7%). Adherence was high with 92.8% EBF until six months and 79% CF thereafter. Only 4.5% had ceased breastfeeding completely at 6 months and 97.8% had ceased breastfeeding by 15 months (Figure 2) (age range of COB 1.5–17 months).

Self-reported adherence to taking cART was unreliable as it correlated poorly with attendance at dispensary visits (data not shown). Thirty-six of 226 women (15.9%) missed at least one dispensary visit (i.e. were greater than two weeks late for a scheduled visit and therefore would have had a drug holiday due to medication shortage). Four of 35 (11.4%) women who missed a visit vs. 5 of 184 (2.7%) women who did not miss a visit transmitted HIV (p=0.038).

### Maternal and infant adverse events

In order to compare adverse events during different time periods, HIV-infected and HIV-uninfected infants are analyzed separately as in previous studies [9]. In HIV-uninfected infants (Table 3), FTT was greatest in the first six months of life (p<0.001), the rates of diarrhoea rose after six months of age (during the period of CF) when compared with the first six months of life (EBF; p<0.001) and the rates of respiratory tract infections rose during the period of COB (12–18 months of age) when compared with the periods of EBF (0–6 months) and CF (6–12 months; p<0.001 for both comparisons). The number of HIV-infected infants was small making estimates of the incidence of adverse effects in this group unreliable (see Supplementary Table 6). Maternal adverse events are in Supplementary Table 7.

**Table 3:** Total infant adverse events (excluding HIV-infected infants)

	Time period^a^				*p* b		
		
Adverse event	0–6 months (n, R)	6–12 months (n, R)	12–18 months (n, R)	0–18 months (n, R)	6–12 vs. 0–6 months	12–18 vs. 0–6 months	12–18 vs. 6–12 months
Diarrhoea	18 (0.36)	55 (1.16)	61 (1.33)	134 (0.94)	0.00002	<0.00001	0.45966
Respiratory infection	12 (0.24)	11 (0.23)	57 (1.24)	80 (0.56)	0.94240	<0.00001	<0.00001
Failure to thrive	88 (1.75)	34 (0.72)	33 (0.72)	155 (1.09)	0.00001	0.00001	0.98412
Rash	71 (1.42)	15 (0.32)	14 (0.30)	100 (0.70)	<0.00001	<0.00001	0.92923
Candidiasis	14 (0.28)	6 (0.13)	0 (0.00)	20 (0.14)	0.11281	0.02262	0.08464
Malaria	6 (0.12)	2 (0.04)	9 (0.20)	17 (0.12)	0.22568	0.35831	0.05407
Nonspecific infection	14 (0.28)	0 (0.00)	3 (0.07)	17 (0.12)	0.02125	0.02517	0.19028
Tuberculosis	0 (0.00)	2 (0.04)	0 (0.00)	2 (0.01)	0.28298	0.96486	0.30926

*Note*: Rates are based on per 100 person-weeks.

a Patients contributed the respective person-time corresponding to each time period.

b *p*-Values were computed using a Poisson regression.

## Discussion

This study provides an estimate of the real-world efficacy of continued maternal cART. The government of Malawi reviewed their experience of the efficacy of Option B+, but HIV status was documented at 12 months or beyond on only 54% of infants leading to an inability to make a reliable estimate of efficacy [23]. Furthermore, a recent review did not find any reports which were able to assess late MTCT through the end of 12 months of breastfeeding using option B or B+ [24]. We believe that our study is the first to report rates of MTCT at 18 months of age.

Predictive models to estimate the efficacy of different national programmes to lead to population wide HIV MTCT rates <5% have suggested that very high population coverage with maternal ARVs will be necessary [25, 26]. Our data showing 4.1% MTCT using continued maternal cART within the context of a clinical study suggest that even at 90% enrolment in ARV programmes nationally, achieving a population wide MTCT rate of less than 5% may be challenging [25]. Furthermore, loss to follow-up antepartum (15%) and postpartum (13%) would have led to a significant number of women discontinuing cART. This occurred despite using adherence counselling, food and transportation stipends and text messaging reminders to enhance follow-up. Therefore, the true intent-to-treat transmission rate would likely be higher than 5% even within this study cohort. Very high “real-world” rates of loss to follow-up in pregnant and breastfeeding women on cART have been found in other studies [27–29] and so we feel that our data reflect real-world efficacy. The 95% CI for the MTCT rate was 2.2–7.6%, and so further studies with larger cohorts will be required to confirm the rate of MTCT using continued maternal cART.

Six episodes of post-partum transmissions occurred and of these, all five for which data were available to determine timing occurred after six months of age. Adherence to maternal cART was poor or questionable in all five women in our cohort who transmitted after six months (Supplementary Table 2). The transmission of a K65R resistance mutation leading to potential failure of future cART in one infant is concerning. A meta-analysis of 51 studies found that only 53% of women had adequate adherence to ARVs in the postpartum period [30].

When analyzed as a continuous variable, CD4 count was not found to be correlated to HIV transmission or infant death in the postpartum period (data not shown). Our low event rate likely led to a lack of sensitivity in identifying a relationship. Only the maternal VL measured at six months was predictive of subsequent HIV transmission. This is consistent with data involving heterosexual transmission of HIV, which demonstrated the importance of viral load in risk of transmission [31]. Our data suggest that measurement of maternal VL at six months of age may help to identify infants at higher risk of late transmission. Patients displaying virologic failure, or patients who have missed dispensary visits, may benefit from further counselling regarding ARV adherence, both for the mother's health and to prevent MTCT although this approach would require further study to confirm. Maternal adherence to treatment may have been improved by using a daily rather than a twice daily cART regimen [32]. It is notable, however, that twice daily ZDV/3TC is still a preferred option in both the US and British treatment guidelines for pregnant women [33, 34].

Our data support the logical premise that poor adherence leading to an elevated maternal viral load appears to be the main factor responsible for post-partum transmission. We base this on the observation that not only elevated maternal viral load but also missed dispensary visits were associated with transmission. However, the hypothesis that there may be something inherently different about the EBF (0–6 months) vs. the CF and COB (6–18 months) periods that makes the latter particularly susceptible to MTCT in the setting of an elevated viral load is also possible. The trend towards a higher transmission rate/100 person-weeks during 6–18 months when compared to 6 weeks to 6 months (p=0.167) is suggestive. In addition, when including transmitting and non-transmitting mothers, more mothers had a detectable VL (>50) at birth than at six months (p=0.002), and mean Log_10_ viral load at six months was not different from that obtained at birth, which would lead one to expect that there would be equivalent or more transmissions in the 0 to 6 months vs. 6 to 18 months period if VL level was the sole factor responsible for MTCT, which was not seen in our results. The mechanism of this potential inherent difference between the EBF (0–6 months) and CF/COB (6–18 months) period remains speculative but may include ingestion of contaminated water, fluids and food, which may lead to gut mucosal injury and disruption of immune barriers which has been proposed for the increased MTCT seen in previous studies [35, 36] that identified a higher transmission rate with mixed feeding compared with EBF. This hypothesis should be interpreted with caution, however, given our low event rate, our VL comparator convenience sample being limited to 96 non-transmitting mothers and that there was a trend but not a statistically significant difference in transmissions in the 0 to 6 months vs. 6 to 18 months time periods.

Our HIV-uninfected infant mortality rate was highest in the 0 to 6 months’ time period (12 deaths) vs. any other time period (8 deaths in 6–18 months time period). Worldwide, a child's risk of dying is highest in the first 28 days of life [37]; our results were consistent with this, with 8 of 12 HIV-uninfected infant deaths in the 0–6 month time period occurring in the first 28 days of life (Supplementary Table 5). In addition, the well-known ‘survivor bias’ would also predict this drop in mortality as time advances. Comparison of mortality rates in a specific time period (i.e. 6–18 months) to a historic time period (i.e. 0–6 months) is biased in the direction of making the later mortality rate appear lower even though it is being compared within the same population. This is because of the premise that the population that is remaining at the later time period is actually a substantively different population by virtue of the fact that infants who have survived >6 months have already been selected out as being healthier/more resistant to mortality. The fact that this survivor bias was not seen in previous studies is interpreted as a complication of the early COB in those studies [9, 12]. By contrast, in our cohort, the total and HIV-uninfected infant mortality rates fell significantly after six months, which suggests a protective effect of on-going breastfeeding.

Our absolute rate of infant mortality in the first six months of life was higher than that seen in the recently published BAN study in Malawi [9]. This difference may reflect the reported higher under-5 mortality rate in Zambia when compared to Malawi, the aetiology of which is probably multifactorial [38]. This fact, however, should not change the within-populations comparison of under and over 28-week death rates seen, which showed a significant increase in mortality after 28 weeks in the Malawi population which utilized early COB (from 0.065 to 0.12/100 person-weeks) vs. a decrease in mortality after 28 weeks in our cohort (from 0.2183 to 0.0755/100 person-weeks; p=0.007). Overall, this would suggest that the difference in infant-feeding practices between the two studies is a very plausible explanation for the demonstrated reversal in pattern of the change in mortality at six months. Nevertheless, comparisons in outcomes between different cohorts need caution in their interpretation.

Our infant FTT was significantly higher during EBF when compared to later time periods (Table 3). We believe that this is also likely due to a survivor bias, and this also impacts early death rates as about 45% of all child deaths are linked to malnutrition [37]. After infants are developmentally advanced enough to accept significant amounts of semi-solid/solid foods (typically after six months), the available options to provide safe, high-caloric and nutrient-rich foods expand significantly.

## Conclusions

Continued maternal cART can achieve the global UNAIDS target of <5% MTCT for eradication of paediatric HIV within the context of a clinical study but loss to follow-up and poor adherence to therapy may limit the benefit. Continued breastfeeding may prevent the rise in infant mortality at six months seen with early COB. These data will help to inform policy decisions regarding implementation of the new WHO guidelines.

## Supplementary Material

Efficacy of WHO recommendation for continued breastfeeding and maternal cART for prevention of perinatal and postnatal HIV transmission in Zambia
