# Supplementary material for: Efficacy of WHO recommendation for continued breastfeeding and maternal cART for prevention of perinatal and postnatal HIV transmission in Zambia
Source: J Int AIDS Soc. 2015 Jul 1;18(1):19352. doi: 10.7448/IAS.18.1.19352 (PMC4490793; doi:10.7448/IAS.18.1.19352)
Supplement: Efficacy of WHO recommendation for continued breastfeeding and maternal cART for prevention of perinatal and postnatal HIV transmission in Zambia [file JIAS-18-19352-s001.pdf]

Supplementary Data/Appendix

Efficacy of WHO recommendation for continued breastfeeding and maternal cART for prevention of perinatal and postnatal HIV transmission in Zambia

Ngoma MS et al.

Supplementary Methods

Both mothers and infants were assessed on each visit up to and including 18 months to collect data through clinical questionnaires as well as blood sampling, which included maternal CD4 counts (Beckman Coulter Epics XL instrument with reagents of Flowcare PLG CD4, Flow-count Fluorospheres, Isoflow Epics Sheath Fluid and Immuno Prep), Hemoglobin measures (Hemocue Hb30 system) and infant heel-prick dried blood spot (DBS) HIV DNA polymerase chain reaction (PCR) testing (Aptima)[19]. A further follow-up visit at 24 months obtained clinical data but not laboratory sampling. Maternal plasma viral loads were assayed on all transmitting mothers and on a convenience sample of 96 non-transmitting mothers at birth and 6 months of age. Cost constraints prevented assessment of all maternal samples for viral load testing. Maternal viral loads were assayed using the Abbott Real Time HIV-1 assay, which was run on the m2000 Real Time System (ABBOTT Laboratories, IL USA). Drug Resistance Genotyping was performed on transmitting mother infant pairs as previously described[20]. Nucleic acids were isolated from mothers' plasma or infants' DBS specimens and purified using the EasyMag system (BioMerieux Canada), an automated guanidium thiocyanate-magnetic silica method. HIV pol was amplified from 10 ul nucleic acid extract using an in house nested reverse transcriptase PCR (RT-PCR) methods targeting HIV pol. The method produced overlapping segments that span protease and the first 900 nucleotides of reverse transcriptase (RT). Sequences were resolved using an ABI Prism 3130xl genetic analyzer. Electropherograms produced were aligned to a reference sequence with SeqScape software (Applied Biosystems,

Canada) and drug resistance genotypes obtained from the HIV Drug Resistance Database (http://hivdb.stanford.edu/).

Supplementary Results

Supplementary Table 1 –Socioeconomic Details of Entire Maternal Study population

Characteristic	N=279
Mean maternal Age (years)	28.6 (SD=5.5)
Maternal parity no/total number (%)	
First child	54/279 (19.3)
Second or third child	139/279 (49.8)
≥4 children	86/279 (30.8)
Death of one or more previous children no/total no (%)	48/279 (17.2)
Marital status- no/total no (%)	
Married	235/279 (84.2)
Single	42/279 (15.1)
Widowed, divorced or separated	2/279 (0.7)
Education- no/total no (%)	
Less than primary	16/278 (5.7)
Primary completed	110/278 (39.6)
Secondary completed	122/278 (43.9)
College/university completed	30/278 (10.8)
Availability of Electricity- no/total no (%)	
None	107/278 (38.5)
Poor/Unreliable	3/278 (1.1)
Fair	21/278 (7.5)
Good	130/278 (46.8)
Excellent	17/278 (6.1)
Mean average monthly household income at enrollment- Kwachas (SD)	731,025 (964,899)

Supplementary Table 2 Details of Transmissions

Patient Number	Time of Transmission (first positive dbs*)	Baseline CD4 (cells/ µL)	Clinical Data
41	Antepartum (birth)	401	Premature labour (33 weeks) after only on treatment for 9 days (incorrect dates of LMP^)
113	Antepartum (birth)	137	Enrolled at 31 weeks, birth at 42 weeks NVD[#]
271	Birth (6 weeks)	79	Enrolled at 24 weeks, delivery at 38 weeks emergency C-section for breech complications. Negative at birth.
33	CF**(12 months)	592	Religious experience of cure at 7 months; stopped medications, but continued breastfeeding; negative at 6 months.
73	CF**(12 months)	225	Began drinking alcohol heavily and stopped regular cART taking. Negative at 6 months.
1	CF**(12 months)	193	Poor Adherence- High Maternal Viral Load throughout protocol. Adherence counsellor suspected she never took her meds (although patient denied this). Negative at 6 months.
18	Wean (18 months)	295	Mother became psychotic and violent. Admitted to hospital. History of weaning unreliable. Stopped taking meds. Infant Negative at 15 months.
32	Wean (18 months)	286	Moved to another district and claimed she was getting medications there. Weaned between 12-13 months. Negative at 12 months
77	Unknown	377	Negative PCR at birth and 2 weeks. Mother defaulted and refused medications or follow-up. Mother then died at 15months postpartum and father presented with child at 18 months requesting follow-up- dbs pos.

*dbs= dried blood spot
^LMP= Last menstrual period
#NVD= Normal Vaginal Delivery
CF**= complementary feeding

Supplementary Table 3: Summary of Resistance Data on Transmitting Mother Infant Pairs

**Patient Number**	**Timing of Sample Tested (Month Postpartum)**			**Resistance mutations**		**comments**
	**Mother**	**Infant**		Reverse Transcriptase	Protease	
**1**	**8months**			**WT**	**WT**	
	12 months			WT	WT	
		18 months		WT	WT	
**18**	**6months**			**WT**	**WT**	
		18months		WT	WT	
**32**	**6months**			**WT**	**WT**	
	9months			WT	WT	
		24 months		WT	WT	
**33**	**3months**			**WT**	**WT**	
	12 months			WT	WT	
						Infant sample not able to be analyzed
**41**	**Birth**			**WT**	**WT**	
		6weeks		K65R, 101EK	WT	
**73**	**6weeks**			**K101E**	**WT**	
	8months			K101E	WT	
		15months		K101E	WT	
**77**						**NA**
**113**		**6weeks**		**WT**	**WT**	
						Mother not available
**271**	**36 weeks antepartum**			**WT**	**WT**	
	Birth			WT	WT	
		6weeks		WT	WT	

*WT= Wild Type, NA= Not available

Supplementary Table 4 Maternal Deaths

Patient Number	Baseline CD4 (cells/ µL)	Cause of Death
140	406	Preeclampsia
151	165	Miscarriage: severe nausea, vomiting, diarrhea and abdominal pain: left hospital against medical advice before investigation and moved out of town
164	62	Stopped medication immediately postpartum due to fears of disclosure. Died at 8 weeks postpartum
77	377	Stopped medications 2 weeks postpartum. Denied adverse effects.<br><br>Moved away. Developed pneumonia, diarrhea and rash then died at 64 weeks

.

Supplementary Table 5 Neonatal and Infant Deaths

Patient number	Age at Death	Cause of Death
21	1 day	Congenital anomalies: cleft palate and hydrocephalus.
46	1 day	Prematurity + Respiratory distress
51	1 day	Birth asphyxia
137	1 day	Chorioamnionitis + Sepsis
166-1	2 days	Sepsis
184	2 days	Perinatal Asphyxia
279	3 days	Umbilical Exomphalos
263	21 days	Crib Death, no warning
41	8 weeks	AIDS
113	8 weeks	AIDS
271	8 weeks	AIDS
181	9 weeks	Pneumonia
239	19 weeks	Malaria

166-2	20 weeks	Failure to thrive + Pneumonia
145	20 weeks	Pneumonia
105	28 weeks	Congenital anomaly of tongue; Surgery- postoperative pneumonia
117	32 weeks	Downs syndrome + Measles + Pneumonia
205	36 weeks	Neck swelling? Lymphoma-
210	12 months	Pulmonary TB + Malnutrition, Weaned at 28 weeks
73	15 months	AIDS
87	15 months	Malaria + Malnutrition
237	15.5 months	Malnutrition+ Pneumonia
33	17 months	AIDS
173	19 months	Malnutrition
67	19.5 months	Mother discontinued meds at 12 months, moved away, food insecure and so continued breast feeding. Cause of death unknown

Supplementary Table 6: Adverse Events in HIV Positive Infants

Adverse Event	Time Period†				P-Value††		
	0-6 Months (n,R)	6-12 Months (n,R)	12-18 Months (n,R)	0-18 Months (n,R)	Overall	6-12 vs. 0-6 Months	12-18 vs. 0-6 Months
Diarrhea	1 (0·60)	1 (0·69)	1 (0·78)	3 (0·68)	0·98149	0·91320	0·84752
Respiratory Infection	0 (0·00)	0 (0·00)	1 (0·78)	1 (0·23)	.	1·00000	.
Failure To Thrive	6 (3·57)	1 (0·69)	0 (0·00)	7 (1·59)	.	0·12949	0·99994
Rash	3 (1·79)	0 (0·00)	0 (0·00)	3 (0·68)	.	0·99996	0·99996
Candidiasis	1 (0·60)	0 (0·00)	0 (0·00)	1 (0·23)	.	0·99998	0·99998
Malaria	0 (0·00)	1 (0·69)	0 (0·00)	1 (0·23)	.	.	1·00000
Nonspecific Infection	0 (0·00)	0 (0·00)	0 (0·00)	0 (0·00)	.	1·00000	1·00000
Tuberculosis	0 (0·00)	0 (0·00)	0 (0·00)	0 (0·00)	.	1·00000	1·00000

Note : HIV Status Was Missing for 12 Infants
Note : Rates Are Based on per 100 Person-Weeks
Note : †Patients Contributed the Respective Person-Time Corresponding to Each Time Period
Note : ††P-Values Computed Using a Poisson Regression

.

Supplementary Table 7: Maternal Adverse Events: n = 226

Symptom	Grading 1-2 (%)	Grading 3-4 (%)
Anemia	123 (54.4)	6 (2.7)
Nausea/Vomiting	35 (15.4)	6 (2.7)
Heartburn	2 (0.9)	0 (0)
Diarrhea	40 (17.7)	16 (7.1)
Candidiasis	0 (0)	7 (3.1)
Cough/Respiratory Infection	26 (11.5)	44 (19.5)
Nonspecific Fever	0 (0)	0 (0)
Malaria	0 (0)	10 (4.4)
TB	0 (0)	4 (1.8)
Hypertension	15 (6.6)	7 (3.1)
Hypotension	2 (0.9)	0 (0)
Rash	45 (19.9)	9 (4.0)
Weight Loss	59 (26.1)	5* (2.2)
Headache	16 (7.1)	23 (10.2)
Vaginal Warts	10 (4.4)	2 (0.9)
Herpes Zoster	0 (0)	3 (1.3)
Herpes Simplex	0 (0)	3 (1.3)
PP** Bleeding	0 (0)	1 (0.4)
PP** Sepsis	1 (0.4)	3 (1.3)

*loss of body weight of 10% or more on 2 separate occasions
** Post Partum
